# Glyphosate and a glyphosate-based herbicide dysregulate the epigenetic landscape of Homeobox A10 (*Hoxa10*) gene during the endometrial receptivity in *Wistar* rats

**DOI:** 10.3389/ftox.2024.1438826

**Published:** 2024-09-13

**Authors:** Virginia Lorenz, Florencia Doná, Dalma B. Cadaviz, María M. Milesi, Jorgelina Varayoud

**Affiliations:** ^1^ Instituto de Salud y Ambiente del Litoral (ISAL), Facultad de Bioquímica y Ciencias Biológicas, Universidad Nacional del Litoral (UNL)–Consejo Nacional de Investigaciones Científicas y Técnicas (CONICET), Santa Fe, Argentina; ^2^ Cátedra de Fisiología Humana, Facultad de Bioquímica y Ciencias Biológicas, Universidad Nacional del Litoral, Santa Fe, Argentina

**Keywords:** glyphosate, endometrial receptivity, Homeobox A10, epigenetics, DNA methylation, histone post-translational modifications

## Abstract

We observed that gestational plus lactational exposure to glyphosate (Gly), as active ingredient, or a glyphosate-based herbicide (GBH) lead to preimplantation losses in F1 female *Wistar* rats. Here, we investigated whether GBH and/or Gly exposure could impair *Hoxa10* gene transcription by inducing epigenetic changes during the receptive stage in rats, as a possible herbicide mechanism implicated in implantation failures. F0 dams were treated with Gly or a GBH through a food dose of 2 mg Gly/kg bw/day from gestational day (GD) 9 up to lactational day 21. F1 female rats were bred, and uterine tissues were analyzed on GD5 (preimplantation period). Transcripts levels of *Hoxa10*, DNA methyltransferases (*Dnmt1, Dnmt3a* and *Dnmt3b*), histone deacetylases (*Hdac-1* and *Hdac-3*) and histone methyltransferase (*EZH2)* were assessed by quantitative polymerase chain reaction (qPCR). Four CpG islands containing sites targeted by B*stU*I methylation-sensitive restriction enzyme and predicted transcription factors (TFs) were identified in *Hoxa10* gene. qPCR-based methods were used to evaluate DNA methylation and histone post-translational modifications (hPTMs) in four regulatory regions (RRs) along the gene by performing methylation-sensitive restriction enzymes and chromatin immunoprecipitation assays, respectively. GBH and Gly downregulated *Hoxa10* mRNA. GBH and Gly increased DNA methylation levels and Gly also induced higher levels than GBH in all the RRs analyzed. Both GBH and Gly enriched histone H3 and H4 acetylation in most of the RRs. While GBH caused higher H3 acetylation, Gly caused higher H4 acetylation in all RRs. Finally, GBH and Gly enhanced histone H3 lysine 27 trimethylation (H3K27me3) marker at 3 out of 4 RRs studied which was correlated with increased *EZH2* levels. In conclusion, exposure to GBH and Gly during both gestational plus lactational phases induces epigenetic modifications in regulatory regions of uterine *Hoxa10* gene. We show for the first time that Gly and a GBH cause comparable gene expression and epigenetic changes. Our results might contribute to delineate the mechanisms involved in the implantation failures previously reported. Finally, we propose that epigenetic information might be a valuable tool for risk assessment in the near future, although more research is needed to establish a cause-effect relationship.

## 1 Introduction

Broad spectrum herbicides such as glyphosate-based herbicides (GBHs) are composed of glyphosate (Gly), its active component, plus undisclosed co-formulants (Mesnage et al., 2019). GBHs are the pesticide most massively used worldwide and ubiquitously detected in the environment and also, in biological samples (Connolly et al., 2020; Grau et al., 2021; Milesi et al., 2021). Studies carried out recently in Canada, United States and Puerto Rico revealed high prevalence of glyphosate (74%–96%) and the aminomethylphosphonic acid (AMPA), its main metabolite (54%–94%) in urine samples of pregnant women predominantly from urban populations (Parvez et al., 2018; Lesseur et al., 2021; Silver et al., 2021; Lesseur et al., 2022; Ashley-Martin et al., 2023). Moreover, these researchers detected positive associations between the concentrations of these compounds and adverse reproductive outcomes such as premature birth (Silver et al., 2021), longer anogenital distance in female infants (Lesseur et al., 2021), and shortened gestational age (Parvez et al., 2018; Lesseur et al., 2022). Recently, the PROTECT study reported correlations between concentrations of AMPA in urine and higher levels of oxidative stress biomarkers in gravid women, particularly at 24–28 weeks of gestation (Eaton et al., 2022). All this evidence indicates that general population, and not only farmers or rural residents, are environmentally exposed to glyphosate and AMPA, and importantly, the health of future generations may be compromised.

International regulatory agencies state that glyphosate herbicide has shown no evidence for being considered as an endocrine-disrupting chemical (EDC) (EPA Environmental protection Agency, 2015; EFSA Europen Food Safety Authority, 2017). However, accumulated data suggests that glyphosate comprise eight of the ten essential traits of an EDC established by the Expert Consensus Statement (La Merrill et al., 2020). One of these characteristics is the ability to trigger epigenetic alterations in cells in charge of hormone production or responsiveness (Muñoz et al., 2021). Epigenetic changes involve molecular factors and processes related to DNA which may have influence over gene expression profiles, without affecting DNA sequence (Beck et al., 2022). The two core types of epigenetic modifications are DNA methylation and hPTMs (such as methylation and acetylation), which are the most studied and best understood (Kumar et al., 2018; Pirola et al., 2018). These modifications are reversible to tolerate transitions between various states against environmental stimuli in order to maintain cellular homeostasis (Zhang et al., 2019). Major epigenetic modifying enzymes, such as DNA methyltransferases (Dnmt3), histone modifiers, among others, play as “writers” and “erasers”, adding or removing different chemical modifications, respectively, regulating this balance (Biswas and Rao, 2018). However, deregulation of epigenetic processes can lead to variations in the expression of the genome or in chromatin structure and, even after withdrawal of the stimulus, promoting the onset and development of different pathological conditions which may be evidenced in the long-term (Holtzman and Gersbach, 2018; Lu et al., 2020). In the last years, several works have shown that both Gly and GBHs have the ability to cause epigenetic changes in different human cell lines and rodent models (reviewed in Rossetti et al. (2021). Nevertheless, there is lack of knowledge to answer whether Gly or GBH formulation is the responsible for the disruption of the different epigenetic mechanisms.

Hoxa10 is a transcriptional factor involved in the regulation of multiple genes associated with embryo implantation not only in rodents but also in humans (Zhu et al., 2013). Hoxa10 interacts with the regulatory sequences of its ensuing destination genes controlling its expression (Vitiello et al., 2008; Bi et al., 2022). At the implantation window, a limited time defined by a receptive endometrium and a competent blastocyst in the course of mid-secretory phase, appropriate expression levels of Hoxa10 are crucial for successful embryo implantation (Guo et al., 2020; Zhao et al., 2021). It has been showed that particular epigenetic mechanisms act in concert to increase *Hoxa10* gene expression and promote endometrial receptivity progression (Zhao et al., 2021). However, there is still the need for further testing to understand *Hoxa10* regulation during the receptive window.

We formerly demonstrated that F1 female *Wistar* rats exposure to a GBH formulation or Gly during the gestation plus lactation period provokes preimplantation losses (Lorenz et al., 2020). Moreover, in that work GBH and Gly disrupted 17β-estradiol serum levels and the expression of uterine markers of endometrial receptivity; which might be linked with the implantation failures detected (Lorenz et al., 2020). In this work, we analyzed the effects of a GBH and Gly on *Hoxa10* gene expression and possible alterations in epigenetic modifications (DNA methylation and hPTMs) in the regulatory regions of this gene, as mechanisms of action of the herbicide during the endometrial receptivity status. Finally, we compare exposure effects to commercial formulation and the active ingredient, Gly.

## 2 Materials and methods

### 2.1 Chemicals

In the present study, we used analytical grade reagents and chemicals. Glyphosate (N-(Phosphonomethylglycine) (CAS Number: 1071-83-6) (purity grade of 96%) was acquired from Sigma-Aldrich Inc. (Saint Louis, MO, United States). The commercial formulation, under the name MAGNUM SUPER II, is an Argentinian brand locally manufactured by Grupo Agros S.R.L. This formulation consists of 66.2% of glyphosate potassium salt (equivalent to 54% w/v/ of glyphosate acid), the active ingredient, in addition to inert components and coadjuvants which are not specified.

### 2.2 Animals

Our experiments were performed with inbred *Wistar*-derived strain rats bred at the Instituto de Salud y Ambiente del Litoral (UNL - CONICET). Animals were housed under guarded environmentally-safe conditions at 22° ± 2°C and 14 h light/24 h day in stainless steel cages with wood bedding. Also, *ad-libitum* access to laboratory chow (16-014007 Rat-Mouse diet, Nutrición Animal, Santa Fe, Argentina) and tap water was guaranteed. All experimental protocols were approved by the Institutional Ethics Committee at the Facultad de Bioquímica y Ciencias Biológicas, Universidad Nacional del Litoral (UNL) in Santa Fe, Argentina (authorization No CE2019/60). We proceeded following the principles outlined in the Guide for the Care and Use of Laboratory Animals issued by the United States National Academy of Sciences.

### 2.3 Experimental design

The experiment was conducted with virgin female rats which were housed overnight with proven-fertility males at the proestrus stage. Coitus was determined the following morning by sperm-positive vaginal smear, designating that day as GD1. Pregnant *Wistar* rats (F0) were transferred to individual cages and ramdomly distributed to one of these regime groups: 1) Control (N = 9), fed with a laboratory pellet chow-based paste, and 2) GBH (N = 8) or 3) Gly (N = 8), fed with a GBH formulation or Gly supplemented paste, respectively. Different types of pastes for each treatment group were prepared according to Milesi et al. (2018) and Lorenz et al. (2020). Shortly, standardized amounts of water and pellet chow (Nutrición Animal, Santa Fe, Argentina) were mixed. In the case of GBH or Gly-groups, a commercial formulation or glyphosate (active ingredient), respectively, was water-diluted and combined with the pellet. Moreover, both the active-ingredient mass and the volume of GBH incorporated into the laboratory paste in each food batch were standardized allowing the accomplishment of similar doses to the ones in our prior woks (Milesi et al., 2018; Lorenz et al., 2020). While the dose administered to the exposed groups was of 2 mg of Gly/kg bw/day, the doses finally reached were 3.8 and 3.9 mg of Gly/kg bw/day for groups GBH and Gly, respectively (difference not statistically significant) considering the weight and the food consumption of F0 dams along the treatment, as reported in Lorenz et al. (2020). These doses are of relevance since they are in the order of magnitude of the chronic reference dose (cRfD) for glyphosate herbicide according to United States Environmental Protection Agency (EPA) (EPA Environmetal protection Agency, 2017). Moreover, the doses are in accordance with the corresponding 1 mg/kg bw/day minimal risk level (MRL) from the Agency for Toxic Substances and Disease Registry (ATSDR) (ATSDR, 2020). Importantly, the paste was made the same day the food was renewed for all groups, i.e., every 3 days. As demonstrated previously, the levels of Gly in the laboratory paste did not change during this period (Milesi et al., 2018). F0 dams received the oral treatment during gestation and lactation, from GD9 until the end of weaning which corresponds to the lactational day 21. The selection of GD9 to initiate the treatment was defined based on 1) the necessity to avoid pregnancy loss during early pregnancy since embryo implantation occurs in the evening of GD5 (Milesi et al., 2015) and 2) the beginning of rat fetal organogenesis. At the time of weaning, one F1 female offspring was randomly selected from each litter in the control, the GBH- and Gly-exposed group to avoid litter effect. Then, F1 female rat reached the sexual maturity and on postnatal day 90 they became pregnant with males of proven fertility. Finally, on the morning of GD5 (corresponding to the preimplantation period) F1 female rats were sacrificed and uterine samples were collected (N = 8–9 animals per experimental group from a different F0 dam). In order to do that, female rats were immobilized by an experienced operator, placed with their heads in an animal guillotine and quickly decapitated. After that, samples were kept in liquid nitrogen and then, transferred at −80°C until use. Regarding F1 male offspring, other experimental assignment was carried out.

Potential confounders of our experimental design were considered, as follows: F0 dams’ food consumption during the exposure period, F0 dams’ weight at GD1, F1 litter size, F1 litter weight at birth, F1 female pup weight at birth, age of F1 female rats at GD1. Importantly, no statistical differences were observed regarding above confounders.

### 2.4 RNA extraction and reverse transcription

Uterine tissue (N = 8–9 animals/group) were collected on GD5 and individually homogenized in TRIzol reagent (Invitrogen, Carlsbad, CA, United States). Then, total RNA was isolated following the manufacturer’s guidelines and the concentration and purity of total RNA were determined by measuring the optical density at 260 and 280 nm. Samples were stored at −80 C until later analysis. The 1 μg of RNA reverse-transcription into cDNA was carried out using Moloney Murine Leukemia Virus reverse transcriptase (10 units; Promega, Madison, WI, United States) using 200 pmol of random primers (Promega). Twenty units of ribonuclease inhibitor (RNAsin; Invitrogen Argentina, Buenos Aires, Argentina) and 100 nmol of a deoxynucleotide triphosphate (dNTP) mixture were added to each tube to a final volume of 30 μL of 1 x reverse transcriptase buffer. Reverse transcription reaction was performed at 37°C for 90 min and at 42°C for 15 min. Reactions were stopped by heating at 80°C for 5 min and cooling on ice. Finally, each reverse-transcribed product was diluted with ribonuclease-free water to a final volume of 60 μL.

### 2.5 qPCR analysis

Reverse-transcribed products (5 µL) were added to HOT FIRE Pol Eva Green ^®^ qPCR Mix Plus (Solis BioDyne; Biocientífica, Rosario, Argentina) and 10 pmol of each primer (Invitrogen) to a final volume of 20 µL. Primers utilized for amplification of ribosomal protein *L19* (housekeeping gene), *Hoxa10*, *Dnmt1*, *Dnmt3a*, *Dnmt3b*, *Hdac-1*, *Hdac-3*, *EZH2* genes are detailed in Table 1. Primer sequences were designed with the Vector NTI Suite Version 6.0 software (InforMax Inc., North Bethesda, MD, United States). Products were amplified employing the Real-Time DNA Step One Cycler (Applied Biosystems Inc., Foster City, CA, United States). After initial denaturation at 95 C for 15 min, the reaction mixture was subjected to successive cycles of denaturation at 95 C for 15 s, annealing at 55°C–60 C for 15 s, and extension at 72 C for 15 s. Each sample was quantified in triplicate. Product purity was confirmed by dissociation curves, and random samples were subjected to agarose gel electrophoresis. Controls containing no template DNA were included in all assays, and these reactions did not yield any consistent amplification. The relative expression levels of each target were calculated based on the cycle threshold (Ct) method (Higuchi et al., 1993). The Ct for each sample was calculated using the Step One Software (Applied Biosystems Inc.) with an automatic fluorescence threshold (Rn) setting. The efficiency of PCR reactions was assessed for each target by the amplification of serial dilutions (over six orders of magnitude) of cDNA of the transcripts under analysis using the StepOne software (Applied Biosystems Inc.). The relative expression level of each target was calculated using the standard curve method (Čikoš et al., 2007). For all experimental samples, the target quantity is determined from the standard curve and normalized to the quantity of *L19* (housekeeping gene). No significant differences in Ct values were detected for *L19* between the experimental groups.

**TABLE 1:** Characteristics of primers and PCR products for gene expression analysis by quantitative PCR.

Gene target	Sense primer (5′–3′)	Antisense primer (5′–3′)	Product length (bp)	Annealing temperature (°C)	GenBank sequence
*Dnmt1*	GGC​AGA​CTC​AAA​CCG​ATC​CC	TGC​CTG​GTG​GTT​CTC​CTC​GT	126	59	NM_053354.3
*Dnmt3a*	GCT​GAA​GGA​CCT​GGG​CAT​CC	GGC​CCC​ACT​CCT​GGA​TAT​GC	152	61	NM_001,003,958.1
*Dnmt3b*	ACC​AGA​GGC​CGC​AGA​TCA​AG	GAG​CCA​TCT​CCA​TCA​TCC​GC	116	59	NM_001,396,349.1
*EZH-2*	GAT​TTT​CCA​GCA​CAA​GTC​AT	AAC​AGT​TTC​ATC​TTC​CAC​CA	114	52	NM_001,134,979.1
*Hdac-1*	CAA​TGA​AGC​CTC​ACC​GAA​TC	TTG​GTC​ATC​TCC​TCA​GCG​TT	112	53	NM_001,025,409.1
*Hdac-3*	CAA​CTG​GGC​TGG​TGG​TCT​AC	CGA​GGG​TGG​TAC​TTG​AGC​AG	108	56	NM_053448.1
*Hoxa10*	GAA​AAC​AGT​AAA​GCC​TCT​CC	ATA​GAA​ACT​CCT​TCT​CCA​GC	148	54	XM_032,906,492.1
*Rpl19*	AGC​CTG​TGA​CTG​TCC​ATT​CC	TGG​CAG​TAC​CCT​TCC​TCT​TC	99	60	NM_031103.1

*Dnmt*, DNA methyltransferase; *EZH-2*, *e*nhancer of zeste homolog 2; *Hdac*, *h*istone deacetylase; *Hoxa10*, homeobox A10; *Rpl19*, ribosomal protein L19.

### 2.6 Bioinformatic analysis of Hoxa10 regulatory regions

The rat *Hoxa10* locus (LOC100911668) was analyzed to identify CpG islands through MethPrimer software (http://www.urogene.org/cgi-bin/methprimer/methprimer.cgi; RRID: SCR_010269). A CpG island was established as a DNA sequence higher than 200 bp containing a percentage of CG higher than 50% and an observed versus expected CG distribution higher than 0.60. *BstU*I (CGCG) sites, recognized by a methylation-sensitive restriction enzyme, were examined within CpG islands and surrounding regions controlled. Also, putative binding sites for TFs were analyzed with PROMO software (http://alggen.lsi.upc.es/cgi-bin/promo_v3/promo/promoinit.cgi?dirDB=TF_8.3; RRID: SCR_016926) (Messeguer et al., 2002). PCR primers were designed using Vector NTI Suite software, Version 6.0 (Infomax Inc., North Bethesda, MD, United States).

### 2.7 DNA methylation-sensitive analysis

To analyze DNA methylation status of relevant *Hoxa10* gene regulatory regions, enzyme-specific restriction sites were assessed through digestion reactions with methylation-sensitive restriction enzymes followed by qPCR technique. Genomic DNA from each uterine sample was extracted by using the phenol/chloroform/isoamyl alcohol method. 1 μg of DNA was digested with 5 units *Sfi*I (GGCCNNNN|NGGCC) (Thermo Scientific) for 15 min at 50 C in order to downsize DNA, and then, purified with the Wizard SV gel and PCR Clean-Up System Kit (Promega, Madison, WI, United States). 1 μg *Sfi*I-cleaved and purified DNA was digested with 10 units *BstU*I (New England BioLab, Beverly, MA, United States) in 1 × enzyme buffer at 60°C, for 1 h. These products of digestion were also purified with the Wizard SV gel and PCR Clean-Up System Kit (Promega, Madison, WI, United States). An optimized qPCR protocol was applied to analyze the relative methylation levels of the different regulatory regions of *Hoxa10* gene, as described in Section 2.5 from *Materials and Methods*. Each sample was quantified in triplicate. Table 2 shows primer sequences designed with Vector NTI Suite Version 6.0 software for DNA Methylation-sensitive analysis. The relative degree of DNA methylation was calculated by Ct values plotted against the log input DNA, yielding standard curves for the quantification of the samples (Čikoš et al., 2007). When a CpG site is methylated, the restriction enzyme *BstU*I is not able to cut it; allowing amplification of the DNA fragment. Importantly, the target quantity is determined from the standard curve and normalized to the quantity of the internal control which is established as a region devoid of *BstU*I sites (not sensitive to enzyme digestion).

**TABLE 2:** Primers and PCR products for epigenetics analysis in the regulatory regions (RRs) of *Hoxa10* gene by quantitative PCR.

Gene target	Sense primer (5′–3′)	Antisense primer (5′–3′)	Product length (bp)	Annealing temperature (°C)
*Hoxa10-*IC	AAC​TCT​TAG​AAA​ATG​ATG​GG	AGG​AAC​AGC​ATC​TTT​CTT​AA	151	52
RR1	ACC​CCA​GCG​AGA​TTC​TTG​GC	TGA​AAT​CAC​TGC​CAA​GGG​GC	208	59
RR2	GCC​CCT​TCC​GAA​AAC​AGT​AA	GAT​TTT​TAC​AGC​GTC​CCC​AC	219	59
RR3	GCA​GAG​AAA​GGC​GTT​AAG​TT	CCT​CCC​AAT​TTA​CAT​TTT​CC	129	54
RR4	CCG​AGA​AAA​CCG​AAT​CCG​AG	CAG​AAG​GAT​GGG​TAC​AGG​CG	105	55

*Hoxa10*, homeobox A10; IC, internal control; RR, regulatory region.

### 2.8 Chromatin immunoprecipitation assay

Chromatin immunoprecipitation (ChIP) analyses were carried out according to Lorenz et al. (2019). Firstly, ∼50 mg of uterine tissue from each sample was submerged in 1% formaldehyde solution and cross-linking was stopped through the addition of 1 M glycine for 5 min. Secondly, tissue was homogenized with RIPA lysis buffer including 1X protease inhibitor (Complete Mini, Protease Inhibitor Cocktail Tablet, Roche Diagnostics GMBH, Germany) and phosphatase inhibitor (Phos-STOP, Phosphatase Inhibitor Cocktail Tablets, Roche Diagnostics GMBH). Thirdly, centrifugation of homogenates was accomplished at 12,000 rpm for 5 min at 4 C and the supernatants were discarded. Subsequently, the nuclei were lysed in SDS lysis buffer which contains proteases and phosphatase inhibitors (as described before), and incubated for 20 min, on ice. Next, samples were individually sonicated on ice by using a Sonic Vibra-Cell™ VCX750 (Sonics & Materials, Newtown, CT, United States) at 30% of power. The fragments of DNA obtained were about 0.5–1.0 kpb. The samples were maximum-speed centrifuged for 10 min at 4 C and supernatants were kept at −80°C until use. After that, 50 µL of Dynabeads^®^ Protein A (Invitrogen) were incubated for 10 min with 2.5 µL of rabbit polyclonal antibody Anti-Acetyl-Histone H3 (H3Ac) (Upstate Biotechnology, Lake Placid, NY, United States) or Anti-Acetyl-Histone H4 (H4Ac) or Anti-trimethyl-Histone H3 (Lys27) (H3K27me3) (EMD Millipore, Darmstadt, Germany). To establish background signal, an equal amount of the specific antibody was replaced with non-immune rabbit serum (negative control). The sonicated chromatin samples were gently incubated with, the Dynabeads^®^ Protein A-antibody complex with rotation at 4 C overnight. DNA-protein complexes were washed and eluted from the Dynabeads^®^ Protein A using elution buffer (100 mM NaHCO_3_ and 1% SDS), and incubated with 0.5 µL of proteinase K 20 mg/mL (Sigma-Aldrich) at 65 C for 2 h to reverse cross-linking. Next, proteinase K inactivation was achieved by incubation at 95°C for 10 min. Sonicated supernatant was introduced in the protocol as an input control and processed in parallel with the immunoprecipitated complexes (IPs) already eluted at the cross-linking reversal step. DNA purification was carried out with a PureLink™ Quick Gel Extraction and PCR Purification Combo kit (Invitrogen). Recovered DNA was quantified and analyzed by qPCR following 2.5 section from *Materials and Methods*. Table 2 shows the primers used to amplify genomic sequences of the regulatory regions of *Hoxa10* gene. The standard curve method was applied by using input DNA serial dilutions to establish the relative amounts of IPs and input DNA. Triplicates of both experimental IPs and input DNA were run. IPs sample values were subtracted the signals obtained with the nonspecific antibody control. Subsequently, results were expressed as a ratio to input DNA.

### 2.9 Statistical analysis

Kruskal–Wallis test was used to analyze data followed by Dunn’s *post hoc* test with Bonferroni correction for intergroup comparisons. Statistical analysis was performed with R software (version 4.2.0) (https://www.r-project.org/). Results are shown as the mean ± SEM. *p*-values < 0.05 were regarded as significant.

## 3 Results

### 3.1 Exposure to Gly and GBH decreases uterine Hoxa10 mRNA transcript levels at the receptive stage

**FIGURE 1:**
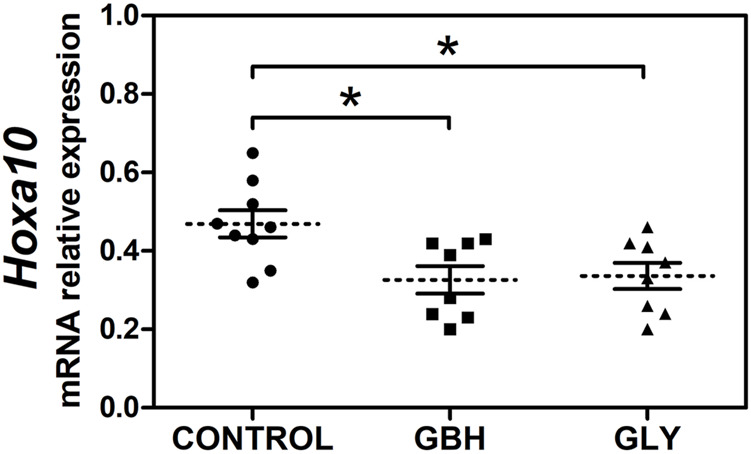
Effect of perinatal exposure to a glyphosate-based herbicide (GBH) or glyphosate (Gly) on the uterine *Hoxa10* gene expression during the receptive stage*.* Relative expression of *Hoxa10* mRNA levels in the uterus of control, GBH- and Gly-exposed F1 female rats on gestational day 5. Results are shown as dots representative of triplicate runs, dashed lines are the mean for each experimental group with the corresponding SEM (N = 8–9 animals/group). Asterisks indicate statistical significance compared to the control: *p < 0.05 vs control (Kruskal–Wallis test followed by Dunn’s *post hoc* test).

First, we evaluated the effect of perinatal (gestational plus lactational) exposure to Gly or GBH on *Hoxa10* gene expression determining the mRNA transcript levels of H*oxa10* from uterine tissue obtained on GD5 (receptive stage). Figure 1 shows that both Gly and GBH exposure caused a decrease in *Hoxa10* mRNA levels compared with control rats. No changes were observed between exposed groups.

### 3.2 In silico detection of CpG islands and relevant TFs for embryo implantation in the rat Hoxa10 gene

**FIGURE 2:**
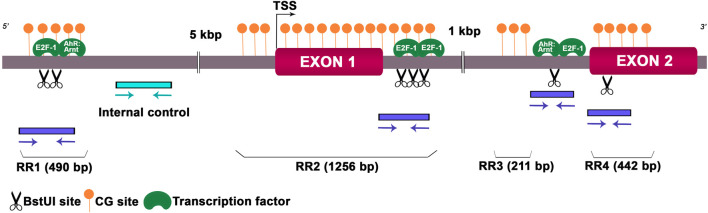
Schematic genomic organization of the rat *Hoxa10* locus. The *Hoxa10* gene comprises EXON 1 and 2 shown by magenta boxes, and the intronic regions between them. *BstU*I restriction sites (CGCG) analyzed are represented by scissor drawings and predicted binding sites for transcription factors E2F-1 and AhR:Arnt are shown in green color. CpG islands named as regulatory region (RR) 1-4 are indicated by filled orange circles. Positions and orientations of the primers to amplify the regions containing the restriction enzyme sites and internal control are indicated by blue and cyan arrows, respectively. TSS, transcription start site.

As we detected deregulated expression levels of *Hoxa10* mRNA in the preimplantation uterus, we wondered whether this gene could undergo epigenetic alterations, including DNA methylation and hPTMs, after Gly and GBH exposure. Along *Hoxa10* locus, we investigated DNA sequences that might be highly susceptible to epigenetic modifications. We detected four CpG islands which included restriction sites for *BstU*I, an enzyme sensitive to methylated CG dinucleotide. We named these CpG island regions as regulatory regions (RRs). Also, we looked for TFs that potentially bind to these specific DNA methylation sites. We identified island 1 (RR1) of 490 bp length including two restriction sites for *BstU*I linked to the AhR:Arnt and E2F-1 TFs, island 2 (RR2) of 1,256 bp length with three restriction sites for *BstU*I linked to the E2F-1 TF, island 3 (RR3) of 211 bp length with a single restriction site for *BstU*I linked to the AhR:Arnt and E2F-1 TFs and island 4 (RR4) of 442 bp length with a single restriction site for *BstU*I and no associated TF. Figure 2 provides a representation of the rat *Hoxa10* gene indicating CG-enriched regions, *BstU*I sites, primer positions to amplify target regions and the binding sites for TFs.

### 3.3 Exposure to Gly and GBH induces DNA hypermethylation in the rat Hoxa10 gene

**FIGURE 3:**
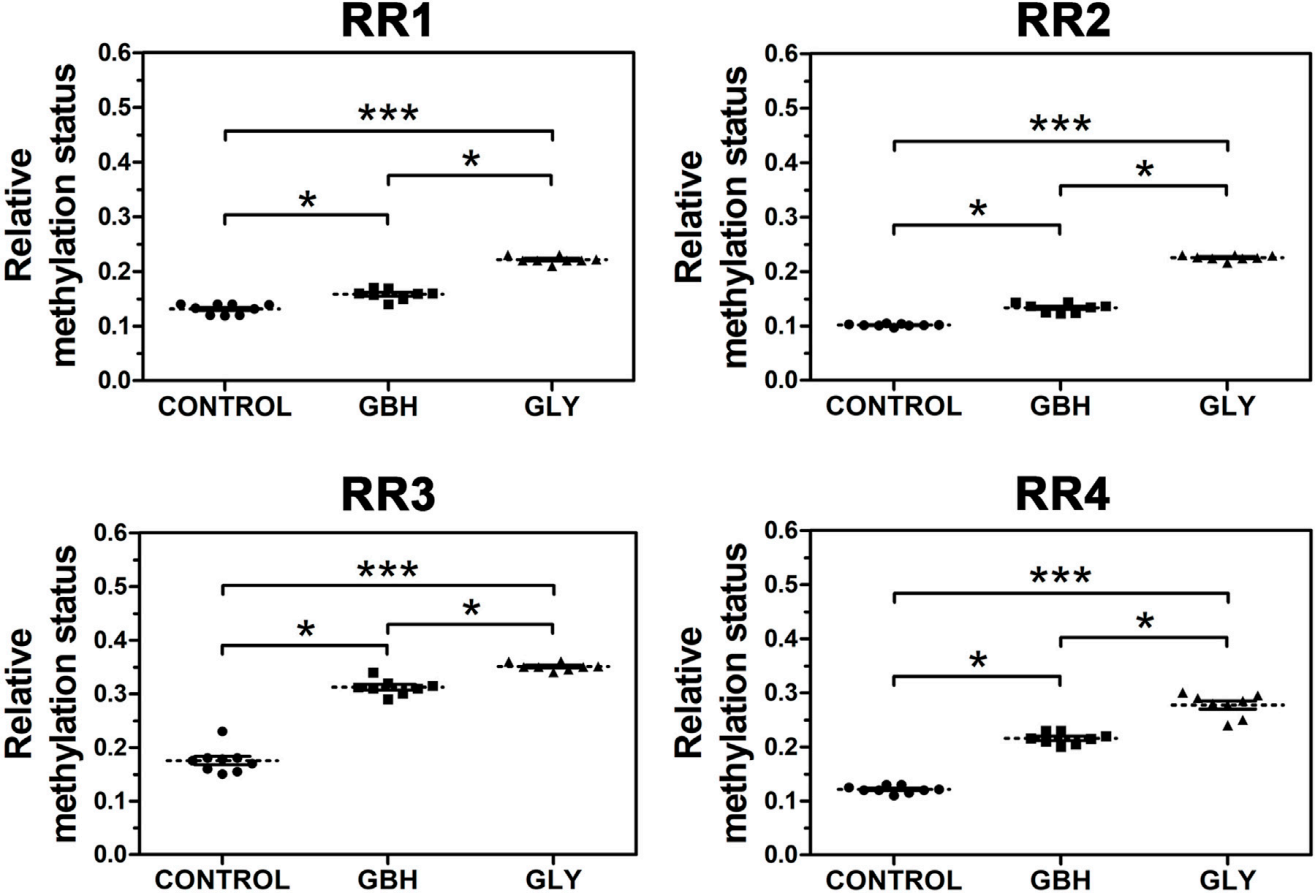
Effect of perinatal exposure to a glyphosate-based herbicide (GBH) or glyphosate (Gly) on DNA methylation status in various regulatory regions (RR) of *Hoxa10* gene. The relative DNA methylation status at specific CG sites was evaluated combining the use of methylation-sensitive restriction enzymes followed by qPCR from uteri on GD5. Results are shown as dots representative of triplicate runs, dashed lines are the mean for each experimental group with the corresponding SEM (N = 8–9 animals/group).Asterisks indicate statistical significance between experimental groups: *p < 0.05; ***p < 0.001 (Kruskal-Wallis test followed by Dunn’s *post hoc* test).

In order to know whether the downregulation of *Hoxa10* mRNA levels correlated with altered DNA methylation patterns, the methylation status of the RRs of this gene was assessed. Uterine genomic DNA samples were subjected to digestion with *BstU*I restriction enzyme and the targeted DNA sequences were analyzed by qPCR. Both Gly and GBH exposure promoted an increased methylation status along the four RRs evaluated in *Hoxa10* gene compared to control group (Figure 3). Moreover, higher levels of DNA methylation were observed in Gly-exposed group than in GBH-exposed group in all the regions evaluated (Figure 3).

### 3.4 Exposure to Gly and GBH causes a differential pattern of hPTMs in the rat Hoxa10 gene

**FIGURE 4:**
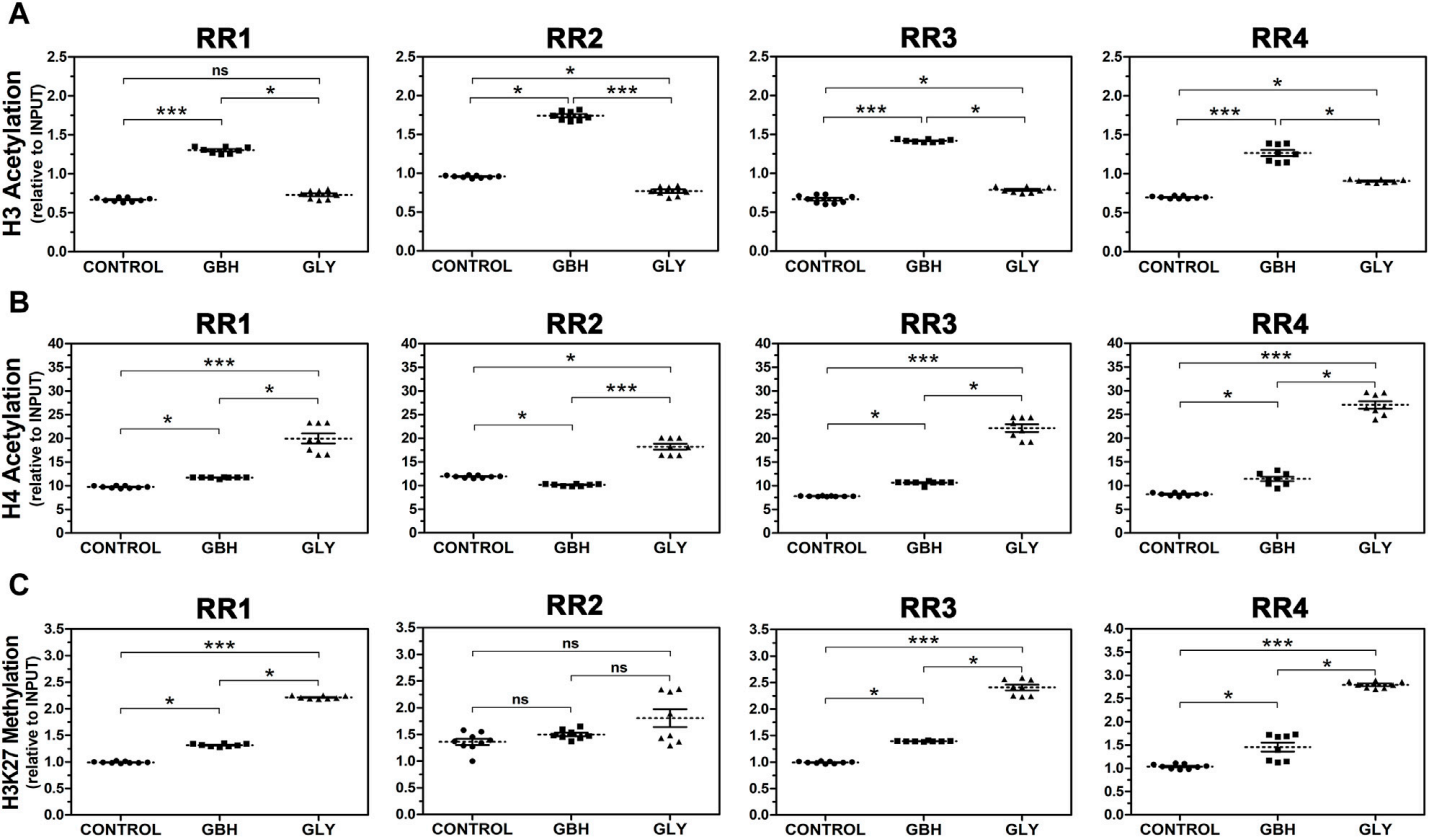
Effect of perinatal exposure to a glyphosate-based herbicide (GBH) or glyphosate (Gly) on the dynamics of histone post-translational modifications in regulatory regions (RR1-4) of the *Hoxa10* gene. **(A)** Histone 3 (H3) acetylation, **(B)** Histone 4 (H4) acetylation, and **(C)** Histone 3 lysine 27 trimethylation (H3K27 methylation) were evaluated from uteri of F1 female rats on GD5 using ChIP assays followed by qPCR. The samples values were normalized to INPUT expression. Results are shown as dots representative of triplicate runs, dashed lines are the mean for each experimental group with the corresponding SEM (N = 8–9 animals/group). Asterisks indicate statistical significance between experimental groups: *p < 0.05; ***p < 0.001; ns, not significant (Kruskal–Wallis test followed by Dunn’s *post hoc* test).

Next, we investigated the abundance of hPTMs in the RRs of the rat *Hoxa10* gene after GBH and Gly exposure by ChIP analyses. Figure 4 shows histone acetylation (H3Ac and H4Ac) and methylation levels at specific lysine residue (H3K27me3) in four regions along *Hoxa10* gene. Regarding H3Ac, all RRs exhibited higher levels of acetylation in GBH-exposed group than in control one. Nevertheless, the effect induced by Gly was different. On the one hand, Gly enhanced acetylation in the RR3 and RR4 compared to control group. On the other hand, Gly reduced acetylation in RR2 compared to control. Moreover, there were differences between the exposed groups in these regions studied. Particularly, in RR1, Gly-exposed group exhibited lower levels of H3Ac than GBH-exposed group with no difference compared to control group (Figure 4A). In relation to H4Ac, the levels of acetylation were increased in GBH-exposed group in RR1, RR3 and RR4 and in Gly-exposed group in all the regions studied compared to the control group. However, in the RR2, GBH induced lower levels of H4Ac compared to control animals. In addition to that, higher levels of H4Ac were observed in Gly than in GBH-exposed group, detecting differences between the exposed groups in all the regions evaluated (Figure 4B). Finally, H3K27me3 was increased in 3 out of 4 RRs studied in both GBH and Gly-exposed groups compared to control group (RR1, RR3 and RR4). In these 3 regions, higher levels of H3K27me3 were determined in Gly-exposed rats in relation to GBH-exposed ones. No changes were detected in RR2 (Figure 4C).

### 3.5 Effect of Gly and GBH exposure on the expression of major epigenetic modifying enzymes

**FIGURE 5:**
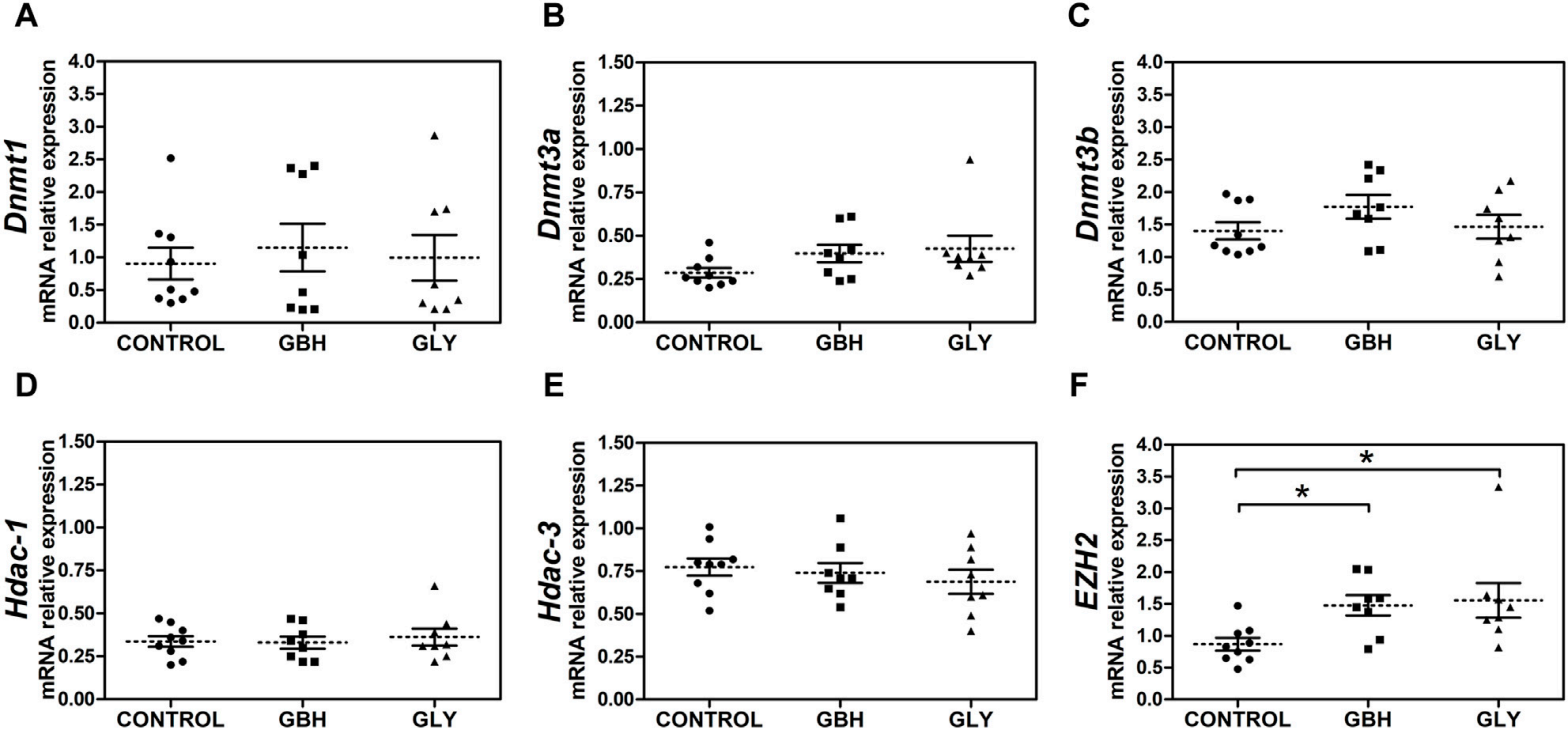
Effect of perinatal exposure to a glyphosate-based herbicide (GBH) or glyphosate (Gly) on major epigenetic enzymes during the receptive stage. **(A)** *Dnmt1*, **(B)** *Dnmt3a*, **(C)** *Dnmt3b*, **(D)** *Hdac-1*, **(E)** *Hdac-3* and **(F)** *EZH2* mRNA relative levels were determined from uteri on GD5 by qPCR. Results are shown as dots representative of triplicate runs, dashed lines are the mean for each experimental group with the corresponding SEM (N = 8–9 animals/group). Asterisks indicate statistical significance compared to the control: *p < 0.05 vs. control (Kruskal-Wallis test followed by Dunn’s *post hoc* test).

Also, we were interested to know whether major epigenetic modifying enzymes accompanied the changes in DNA methylation and hPTMs as key makers indicative of an altered epigenetic state. Firstly, expression levels of *Dnmt3a* showed a trend toward an increase in GBH- and Gly-exposed groups in relation to control group (Figure 5B; *p* = 0.0510), while there was no difference when comparing the exposed groups. As regards *Dnmt1* and *Dnmt3b*, no changes were detected between the experimental groups (Figures 5A, C). Similarly, the transcript levels of *Hdac-1* and *Hdac-3*, enzymes characterized by a highly ubiquitous pattern of expression, showed no changes between experimental groups (Figures 5D, E). Regarding enhancer of zeste homolog 2 (*EZH2*), an enzyme which specifically catalyzes the incorporation of a methyl group on Lys27 in H3, higher expression was detected in GBH- and Gly-exposed groups in relation to control group (Figure 5F).

## 4 Discussion

Previously, we showed that gestational plus lactational exposure to Gly technical grade or a GBH formulation induced implantation failures in F1 female *Wistar* rats (Lorenz et al., 2020). In the current study, we evaluated whether *Hoxa10*, a critical implantation gene, display epigenetic modifications that could be associated with its transcriptional changes during endometrial receptivity. We detected a downregulation of *Hoxa10* transcript levels in the uterus of female rats during the preimplantation period in both GBH- and Gly-exposed groups. It is known that Hoxa10 has a restricted spatio-temporal expression in endometrium with a surge during the window of implantation (Taylor et al., 1998). Female mice with disrupted Hoxa10 expression are unable to sustain embryo implantation (Benson et al., 1996). Additionally, altered uterine Hoxa10 expression have been detected in women diagnosed with endometrial reception disorders such as endometriosis (Özcan et al., 2019; Liu et al., 2021), adenomyosis (Hiraoka et al., 2023), polyps and leiomyomas (Munro, 2019). Taken together, this data supports the fact that lower levels of *Hoxa10* have a negative impact on endometrial receptivity, which might have related with the GBH and Gly-induced preimplantation losses.

Multiple epigenetic modifications have been detected during uterine receptivity (DNA methylation, hPTMs, and non-coding RNAs with assistance of major epigenetic enzymes) (Kong et al., 2019). Particularly, DNA methylation consists of the covalent addition of a methyl group to the fifth carbon of a cytosine base adjacent to a guanine nucleotide (Villicaña and Bell, 2021). Interestingly, we detected a state of DNA hypermethylation in all the RRs studied of the *Hoxa10* gene in both GBH- and Gly-exposed groups. Moreover, slightly higher levels of DNA methylation were detected in rats exposed to the active ingredient alone in comparison to the GBH exposed ones in all the regions evaluated. As high levels of DNA methylation in the regulatory regions of genes are often linked to transcriptional silencing (Jones, 2012), these results correlate with the decreased transcript levels of *Hoxa10*. Other studies showed that Gly or/and GBH cause alterations on global DNA methylation (Woźniak et al., 2020; Ergun and Cayir, 2021; Mesnage et al., 2022) and gene-specific DNA methylation of promoter regions (Lorenz et al., 2019; Woźniak et al., 2020) in various organs and cell cultures. These findings indicate that changes in DNA methylation compromise a mechanism of action of the herbicide. For example, Woźniak et al. 2020 determined decreased global DNA methylation and, particularly, increased methylation patterns in the promoter sequences of TP53 tumor suppressor gene in human blood mononuclear cells. A previous work from our lab detected that estrogen receptor alpha gene was upregulated in the uterus in association with DNA hypomethylation after perinatal exposure to a GBH (Lorenz et al., 2020). Based on the above evidence, GBH and Gly are able to induce different changes in the patterns and/or abundance of DNA methylation depending on genome region or cell type which lead to different effects on gene expression.

*Hoxa10* gene presents putative binding sites for the aryl hydrocarbon receptor (AhR), the aryl hydrocarbon receptor nuclear translocator (Arnt), its heterodimerization partner, and E2F-1 TFs inside RR1 and RR2 and in the vicinity of RR3. The proteins of both AhR and Arnt show uterine specific expression during the peri-implantation period in mouse (Kitajima et al., 2004) and rabbits (Hasan and Fischer, 2001). Moreover, E2F-1 and Arnt were identified as putative TFs during the window of implantation in humans (Tapia et al., 2011). Methylated residues can hinder TF binding leading to repressed transcriptional activity (Tate and Bird, 1993). So, the active role of these TFs during embryo implantation and the hypermethylation of *Hoxa10* suggest that the RRs evaluated and the TFs associated could be checkpoints of transcriptional regulation of this gene after GBH and Gly exposure.

The hPTMs are epigenetic modifications that produced different responses on the regulation of transcription. Some changes on hPTMs detected in our study have been associated with the repression of transcription. Interestingly, although H3Ac and H4Ac favor DNA unwrapping, the effect of Lys acetylation on histone is not cumulative. Both *in vitro* and functional studies (transcription factor binding assays and gene expression analysis) showed that in presence of acetylated H3, H4Ac counteracts the effects of H3Ac (Kurdistani et al., 2004; Agricola et al., 2006; Gansen et al., 2015). In the present work, increased levels of H3Ac were observed in the four RRs studied in GBH-exposed rats in comparison to control and Gly ones. Simultaneously, increased H4Ac occurred in three of these RRs in GBH rats, which might induce an opposite effect respect to H3Ac. When analyzing Gly-exposed rats, we detected co-existence of increased levels of H3Ac in comparison to control in two RRs analyzed with increased levels of H4Acrespect to control group. So, in the Gly-exposed group, H4Ac could also have a modulatory effect on H3Ac, preventing chromatin relaxation in these RRs of *Hoxa10* gene (Gansen et al., 2015). Particularly, in RR2 H4Ac was increased in the Gly group with decreased H3Ac levels. According to Gansen et al. (2015) acetylated H4 alone is able to form more stable nucleosomes. On the contrary, in RR2 H4Ac was decreased in the GBH group with increased H3Ac levels, so the local effect of this combination of histones might be different to the global effect.

Regarding H3K27me3, enriched levels were detected in most of the RRs analyzed in both GBH and Gly-exposed animals with the exception of RR2. Furthermore, higher expression of EZH2 gene, the chromatin-remodeling enzyme which catalyzes H3K27me3, was detected in both GBH- and Gly-exposed groups, correlating with the increased levels of H3K27me3. Given the known relationship of H3K27me3 epigenetic mark with transcriptionally repressed chromatin (Önder et al., 2015), all these results are in concordance with the diminished *Hoxa10* levels. Also, it would be possible the coexistence of H3K27me3 with other repressed marks which could affect *Hoxa10* gene downregulation.

Dnmts, a family of enzymes which catalyzes DNA methylation has been affected by this herbicide. Two of them, *Dnmt*3a and *Dnmt*3b, showed increased transcript levels in liver and kidney from female rats exposed chronically to a GBH formulation (0.1 μg/g) during 2 years (Mesnage et al., 2015; Ergun and Cayir, 2021). In our experimental model, although no differences were detected in the mRNA levels of *Dnmt*1 and *Dnmt*3b, *Dnmt*3a exhibited a trend toward an increase in both GBH- and Gly-exposed rats in comparison to controls (*p* = 0.0510).

According to up-to-date studies, the epigenome may be affected by environmental factors, including environmental EDCs (Kunysz et al., 2021; Nilsson et al., 2022). Chemicals which influence hormone function have identifiable key characteristics associated with their ability to interact with critical regulatory pathways of hormone systems and these attributes can be useful to recognize EDCs (La Merril et al., 2020). One of these characteristics includes the capability to trigger epigenetic changes in hormone-producing or responsive cells for which there are still no standardized tests endorsed by official guidelines (La Merril et al., 2020). As regards Gly herbicide, many regulatory agencies established there is no convincing evidence of potential interplay between Gly and endocrine pathways (EPA Environmental protection Agency, 2015; APVMA, 2016; EFSA European Food Safety Authority, 2017). In this sense, the current study adds information about the potential of both Gly and a GBH formulation to cause different types of epigenetic modifications in the uterus of rats, a highly hormone-regulated tissue by sex steroids. Therefore, we could reaffirm that the environmental pollutant Gly exhibits a key property of an endocrine disruptor (Reviewed in Muñoz et al., 2021).

The present and previous results of our group indicated that Gly and GBH showed similar effects in most of our evaluations; and, importantly, the same chemical compounds were used in both studies (Lorenz et al., 2020). However, some differences on the epigenetic changes were detected between GBH and Gly exposed animals which could be assigned to the presence of certain co-formulants. Until now, few works were dedicated to study the effect of Gly and GBH formulations simultaneously on epigenetic targets (Smith et al., 2019; Mesnage et al., 2022). Mesnage et al. (2022) compared the effects of Gly and a GBH formulation (Roundup Bioflow) on DNA methylation profiling from liver of female adult rats. Although no difference was detected in the percentage of methylated CpG residues, both Roundup Bioflow and Gly induced differentially methylated CpG sites. While Roundup Bioflow caused greatest changes at gene expression and phenotypic level, Gly induced a higher number of differentially methylated CpG sites, suggesting different mechanisms of action of these compounds. However, the differentially methylated sites could not be associated with changes in specific gene expression. Therefore, we highlight the importance of the comparative studies to evaluate whether glyphosate in association with certain co-formulants or the active ingredient alone produce the effects of the herbicide.

**FIGURE 6:**
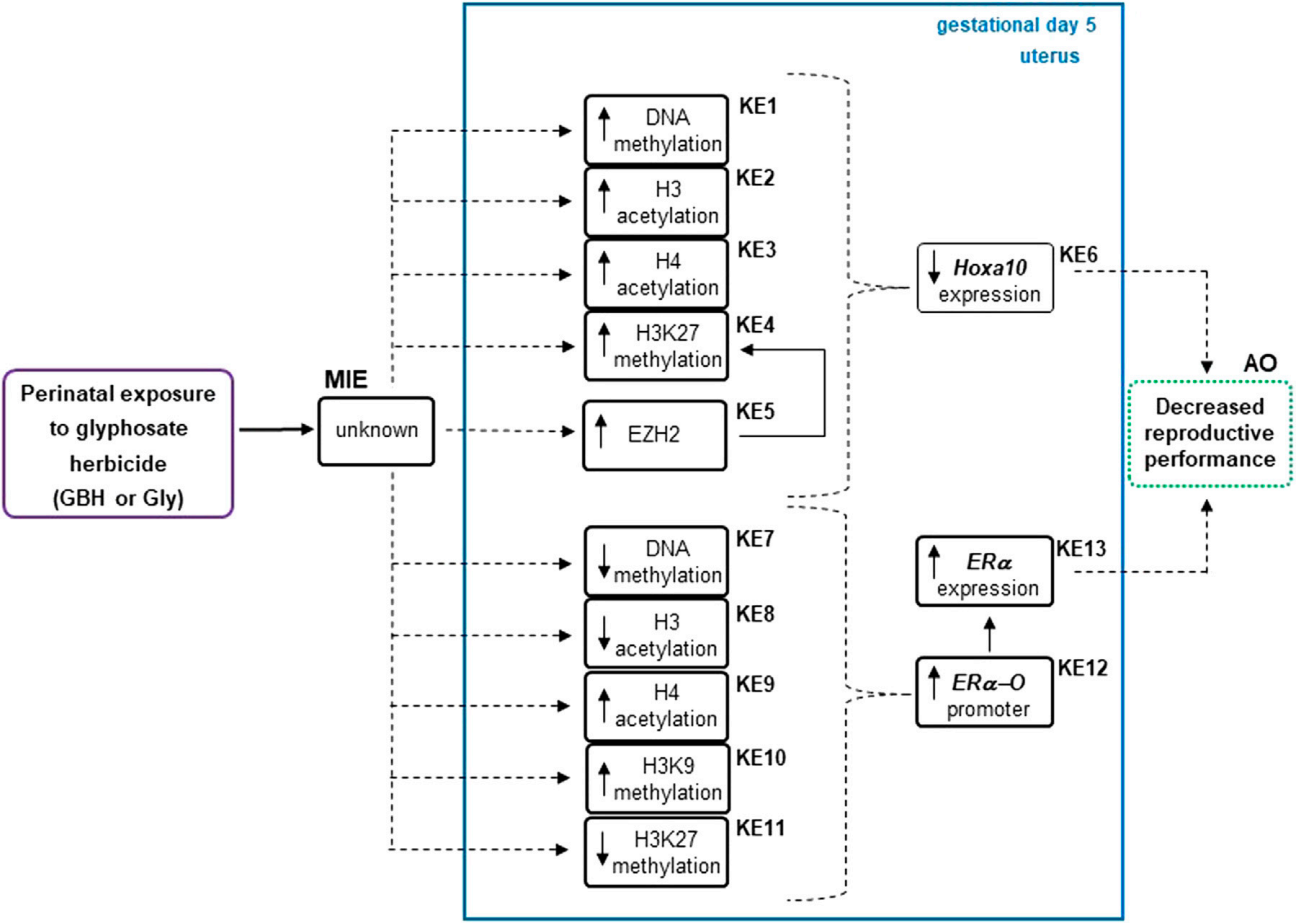
Analysis describing how epigenetic modifications respond to the glyphosate herbicide using an adverse outcome pathways (AOPs) framework. Epigenetic changes as key events (KEs) were detected in the regulatory regions of uterine estrogen receptor alpha (ERα) (Lorenz et al., 2019) and *Hoxa10* gene of *Wistar* rats during the endometrial receptive stage (gestational day 5) after glyphosate herbicide exposure. The up and down arrows within a KE box denote the direction of that change. AO, adverse outcome; EZH2, enhancer of zeste homolog 2; GBH, glyphosate-based herbicide; Gly, glyphosate; H3, histone 3; H3K27me3, H3 lysine 27 trimethylation; KER, key event relationship in solid lines, possible KER in dashed lines, MIE, molecular initiating event.

Organizing epigenetic data into adverse outcome pathways (AOP) frameworks could be useful for identifying cause-effect relationships, knowing how a system will react to a given environmental toxicant and distinguishing possible gaps to delineate future research (Angrish et al., 2018). Figure 6 shows the analysis of present and previous data from our laboratory (Lorenz et al., 2019) on epigenetic changes induced by Gly herbicide in genes involved in the implantation process using AOP framework. We consider this information adds evidence in the direction that there would be a possible relationship between epigenetic modifications and the decrease in the reproductive performance in rats exposed to GBH or Gly (Milesi et al., 2018; Lorenz et al., 2020). However, more research is needed to establish a cause-effect relationship. Considering several epigenetic biomarkers have been used for diagnosis and risk stratification in certain diseases (Jung et al., 2020; Schröder et al., 2022). Epigenetic data might be taken into account for risk assessment in the near future.

In summary, GBH and Gly altered the epigenetic landscape of *Hoxa10* gene inducing changes in the levels of DNA methylation and histone acetylation (H3Ac and H4Ac) and methylation (H3K27me3). Also, EZH2 major epigenetic enzyme resulted affected by GBH and Gly exposure. Finally, we report that Gly and GBH depressed *Hoxa10* mRNA levels through subtly different mechanisms of action.

## Data Availability

The original contributions presented in the study are publicly available in Digital CONICET, the Institutional repository of CONICET. This data can be found here: http://hdl.handle.net/11336/243677.

## Abbreviations

AMPA, aminomethylphosphonic acid; ChIP, chromatin immunoprecipitation; CpG, cytosine-phosphate-guanine dinucleotide; EDC, endocrine-disrupting chemical; EZH2, enhancer of zeste homolog 2; GBH, glyphosate-based herbicide; GD, gestational day; Gly, glyphosate active ingredient; H3K27me3, histone H3 lysine 27 trimethylation; Hoxa10, Homeobox A10; hPTMs, histone post-translational modifications; IPs, immunoprecipitated complexes; qPCR, quantitative polymerase chain reaction; TFs, transcription factors.
